# Anatomic considerations of inflatable penile prosthetics: lessons gleaned from surgical body donor workshops

**DOI:** 10.1038/s41443-023-00715-3

**Published:** 2023-05-13

**Authors:** D. Osmonov, S. K. Wilson, T. Heinze, M. Heimke, J. Novak, A. Ragheb, T. Köhler, G. Hatzichristodoulou, T. Wedel

**Affiliations:** 1 Department of Urology and Pediatric Urology, University Hospital Schleswig Holstein, Campus Kiel, Kiel, Germany; 2 Institute of Urologic Excellence, La Quinta, CA USA; 3 Institute of Anatomy, Center of Clinical Anatomy, Kiel University, Kiel, Germany; 4 Department of Urology, General University Hospital and First Faculty of Medicine, Charles University, Prague, Czech Republic; 5 Department of Urology, Faculty of Medicine, Beni-Suef University, Beni-Suef, Egypt; 6 Mayo Clinic College of Medicine, Rochester, MN USA; 7 Department of Urology, Martha-Maria Hospital, Nurenberg, Germany

**Keywords:** Anatomy, Sexual dysfunction

## Abstract

Surgical implantation of an inflatable penile prosthesis (IPP) remains the gold-standard treatment for severe erectile dysfunction. The ideal surgical technique requires a thorough understanding of the relevant anatomy. This includes anatomic considerations related to, but not limited to, dissection and exposure of penoscrotal fasciae and tissues, corporal configuration, and abdominal structures. Insights obtained from pre-dissected anatomic specimens can obviate urethral injury, nerve damage, corporal perforation, inappropriate sizing, crossover, or implant malposition. We present penile implant-specific anatomic dissections and topographic landmarks identified over the last decade in the course of surgical training programs provided for IPP implantation.

## Introduction

Implantation of an inflatable penile prosthesis (IPP) is the definitive treatment of erectile dysfunction unresponsive to conservative and non-invasive treatment options [1]. Despite high satisfaction rates in patients and partners alike (80.9% and 65.0%, respectively), surgical complications occur regardless of the surgeon’s experience level [2, 3].

During IPP implantation, several anatomical structures are at risk of intraoperative injury [3, 4]. Thorough knowledge of the pertinent anatomy is an essential prerequisite to prevent inadvertent damage [4–10]. The renowned Austrian anatomist Werner Platzer (1929–2017) stated that “anatomy without clinic is dead, clinic without anatomy is deadly” [11]. In accordance with these principles, we established an annual penile implant-specific surgical training program at the center of clinical anatomy at Kiel University and the department of urology at the University Hospital Schleswig-Holstein, Campus Kiel, Germany.

Over the last decade, we have provided an educational platform for prosthetic urologists to review essential anatomic landmarks on pre-dissected anatomic specimens related to IPP implantation and train the stepwise surgical procedure in body donors. In this report we present anatomic dissections obtained from surgical training courses, with the aims of highlighting the topographic anatomy and optimizing IPP outcomes.

## Material and methods

### Body donors

Body donors were recruited from the body donation program at the Institute of Anatomy, Kiel University, Germany. Written consent was obtained for educational and research purposes. Body donors with known previous diseases that would affect the relevant anatomy, malformations, or surgical interventions in the pelvic region and external genitalia were excluded. Pre-dissected anatomical specimens were created from body donors perfused with a solution containing 3% formalin complemented with 75% ethanol and 8% glycerol via femoral arteries, and post-fixed in 70% ethanol. Body donors used for surgical training of IPP implantation were perfused with a solution containing 70% ethanol, 30% glycerol and 0.3% lysoformin via femoral arteries, and stored in a humid atmosphere (1% thymol) before use [12].

### Dissection of anatomical specimens for educational purposes

Dissection of anatomical specimens was performed by clinical anatomists, professional dissectors, and experienced high-volume IPP implanters. The regions of interest were exposed to demonstrate relevant anatomical landmarks as well as adjacent anatomical structures at risk of damage during IPP implantation. The specimens were photo documented (Sony Alpha 7.III, Sony FE 90 mm F2.8 Macro G OSS lens, Sony Remote Version 1.4.00.01241, Japan) for educational purposes. Structures of interest were highlighted in semitransparent colors using Adobe Photoshop (Version 24.0.1, California).

### Surgical training of IPP implantation in body donors

During body donor workshops (*n* = 10, 5 body donors per workshop) the previously described technique [13, 14] of penoscrotal implantation of IPP (Titan® Touch, Coloplast, Denmark, USA, France) was first carried out at a master table in a stepwise procedure, and then performed by the attendees at their own working stations. Prior to the surgical intervention, pre-dissected anatomical specimens were presented to illustrate the topographic anatomy related to each surgical step and point out potential pitfalls.

Photographs and video clips compiled from surgical procedures performed by the authors (DO, SKW) were shared with the attendees. Selected photographs were used to correlate the anatomic dissections and landmarks with the surgical steps and safety checks required during IPP implantation.

## Results

### Penoscrotal access

#### Surgical procedure

**Fig. 1:**
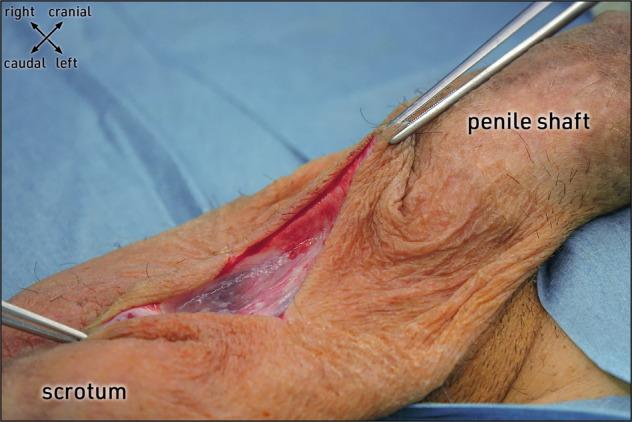
Stepwise surgery: scrotal skin incision. Latero-ventral view of the external genitalia. The penile shaft is reflected cranially, the scrotum caudally. A transverse skin incision is performed about 2 cm below the penoscrotal junction at the upper scrotal region. Image obtained during surgery.

Penoscrotal implantation of an IPP starts with a skin incision performed in either transverse or vertical direction. If a transverse approach is chosen, we recommend a skin incision not directly along the penoscrotal junction but 2 cm below, at the upper scrotal skin region, to prevent postoperative maceration and dehiscence (Fig.1).

#### Topographic anatomy

**Fig. 2:**
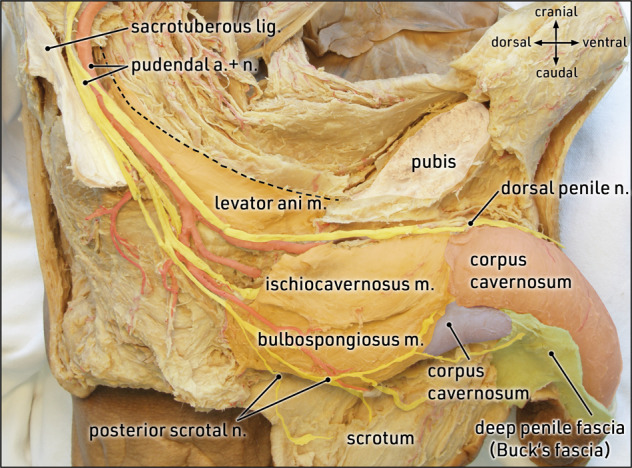
Topographic anatomy: neurovascular supply to the external genitalia. Lateral view of a left hemipelvis (parasagittal section) after exarticulation of the right hip bone. The right-sided levator ani muscle is transected (dotted line) from its origin along the tendinous arch; adipose tissue of the ischioanal fossa is removed to expose neurovascular supply to the external genitalia via branches of the pudendal nerve and vessels. The posterior scrotal and perineal branches divide early in the ischioanal fossa to reach the scrotal skin, the perineal musculature, and muscles covering the corpora cavernosa and spongiosum. The dorsal penile nerve follows the inferior pubic ramus towards the pubic symphysis and extends along the dorsal penile shaft. Formalin-fixed specimen.

**Fig. 3:**
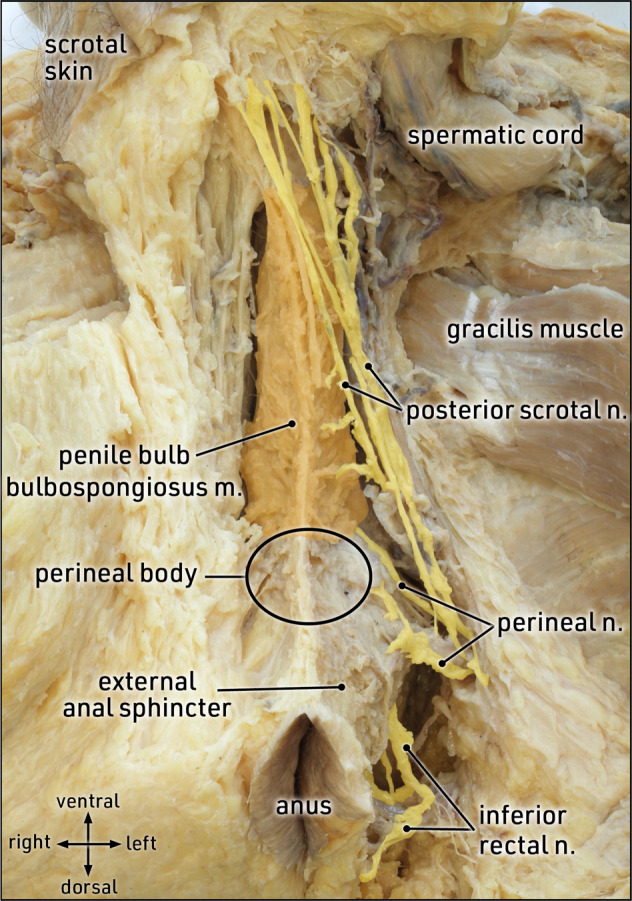
Topographic anatomy: scrotal branches of the pudendal nerve. Caudal view of the perineal region. On the left side, adipose tissue is removed to expose the external anal sphincter, the perineal body, the penile bulb, and branches of the pudendal nerve. The posterior scrotal branches approach the dorsal aspect of the scrotum (reflected cranially) from dorso-lateral to ventro-medial. Formalin-fixed specimen.

The nerves in this region should be considered in order to avoid scrotal skin denervation. Sensory perception in the scrotal skin is mainly provided by the pudendal nerve which originates from the sacral spinal nerves (S2–S4), enters the ischioanal fossa, and gives off several branches to the perineal region, such as inferior rectal nerves, perineal nerves, dorsal penile nerve, and posterior scrotal nerves (Fig. 2). The posterior scrotal nerves approach the scrotal skin from lateral to medial and dorsal to ventral (Fig. 3). Thus, the skin incision must be performed along the central and cranial part of the scrotum, and neither lateral nor caudal, in order to prevent damage to the posterior scrotal nerves.

### Exposure of corpora cavernosa and dissection of penile fasciae

#### Surgical procedure

**Fig. 4:**
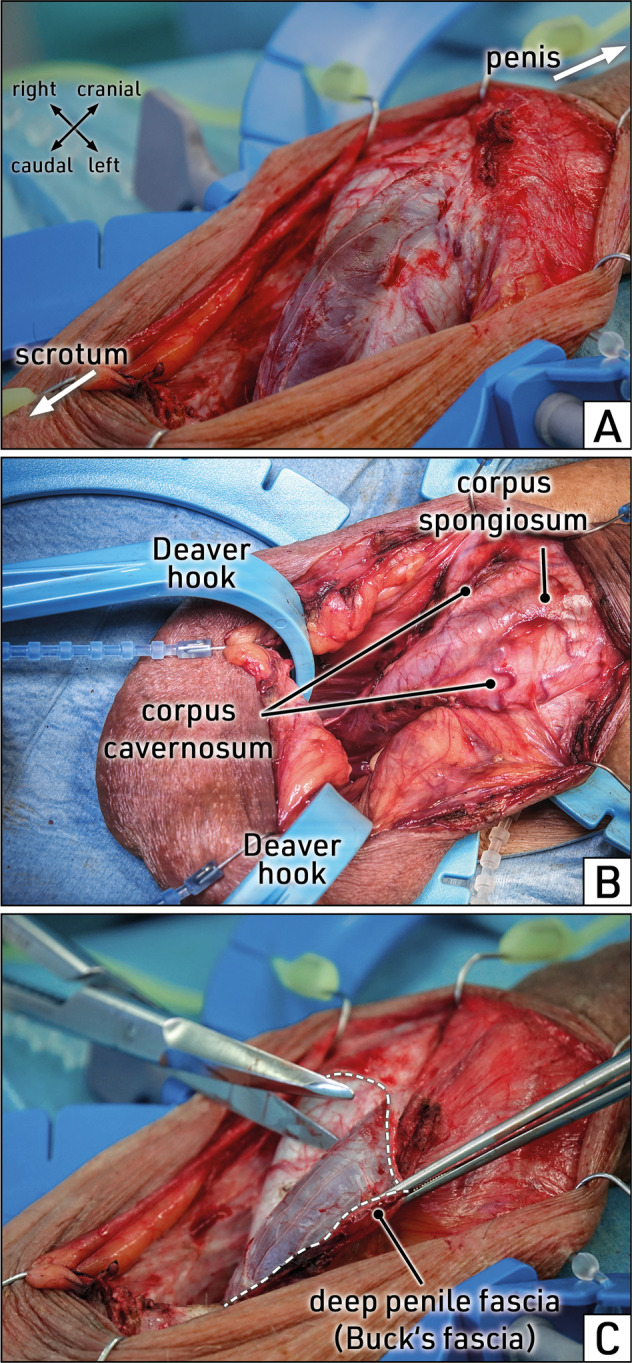
Stepwise surgery: exposure of the corpora cavernosa. Latero-ventral view of the external genitalia. The penile shaft is reflected cranially, the scrotum caudally. **A** The transverse skin incision below the penoscrotal junction is opened further to expose the corpora cavernosa and corpus spongiosum (urethral bulb) covered by penile fasciae. **B** The proximal corpora cavernosa are completely exposed with a Deaver Baby hook. **C** The deep penile fascia (Buck´s fascia) is incised (dotted line) and dissected from the corpora cavernosa in the region of the planned corporotomy. Images obtained during surgery.

A transverse skin incision of about 3 cm allows optimal exposure of the proximal segments of the corpora cavernosa as well, in which cylinder insertion may be difficult due to the conical narrowing of the corpora cavernosa (Fig. 4A). Full exposure and access to this surgical site is best achieved by application of the disposable SKW or the Wilson retractor and Deaver Baby hooks (Fig. 4B). This technique facilitates the implantation of an IPP and reduces operating time because it provides a full view of the region of interest.

Prior to corporotomy, the surrounding area must be freed of overlying tissues comprising the superficial penile fascia (Colles´ fascia) and the deep penile fascia (Buck’s fascia). Incision of the penile fasciae is performed longitudinally in the penile midshaft, parallel and at least 0.5 cm distant from the penile urethral bulb/corpus spongiosum (Fig. 4C). In cases of revision surgery or device explantation, the course of the urethral bulb cannot always be identified; a Babcock forceps should be used to carefully elevate and protect the urethral bulb from inadvertent injury.

#### Topographic anatomy

Dissection and removal of both penile fasciae at the site of the planned corporotomy is essential to expose the “naked” tunica albuginea. In the midshaft of the penis, the penile fasciae are thinner in the ventral paraurethral region than at the dorsal aspect of the penis. When performing a routine IPP implantation, the superficial and deep penile fascia may be dissected together from the corporal tunica albuginea. However, in patients undergoing complex penile reconstruction, the two penile fasciae must be differentiated during dissection of the dorsal penile neurovascular bundle (NVB).

**Fig. 5:**
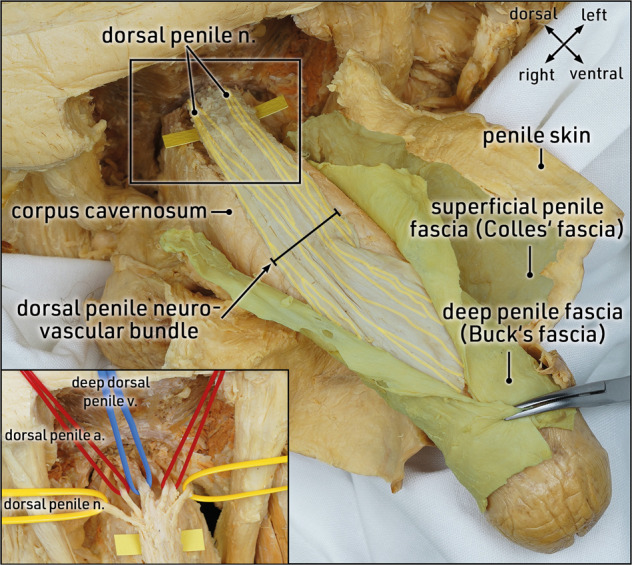
Topographic anatomy: dorsal penile neurovascular bundle, superficial and deep penile fasciae. Cranial view of the penis. The penile skin, the superficial penile fascia (Colles’ fascia, dark green) and the deep penile fascia (Buck’s fascia, light green) are incised along the midline and reflected laterally to expose the dorsal penile neurovascular bundle. The neurovascular bundle forms a condensed trunk at the infrapubic region and spreads out along the dorsal aspect of the penis towards the glans penis in a horsetail-like pattern. The insert demonstrates the main components of the dorsal penile neurovascular bundle at the infrapubic region, consisting of dorsal penile nerves (yellow vessel loops), dorsal penile arteries (red vessel loops), and the deep dorsal penile vein (blue vessel loop). Formalin-fixed specimen.

The dorsal penile NVB adheres to the deep penile fascia and is composed of dorsal penile nerves, dorsal penile arteries, and the deep dorsal penile vein extending dorsally along the penile shaft towards the glans penis (Fig. 5). In the infrapubic region the proximal NVB forms a trunk; laceration of these densely packed nerve fiber bundles will leave most of the glans penis without sensation. However, distally the nerve fibers spread out like a horse’s tail. Injury at this site will result in hypesthesia in a small area. Especially when performing corporotomy during infrapubic IPP placement, the peculiar topographic anatomy of the proximal dorsal penile NVB must be considered in order to prevent permanent denervation of the glans penis.

### Corporotomy

#### Surgical procedure

**Fig. 6:**
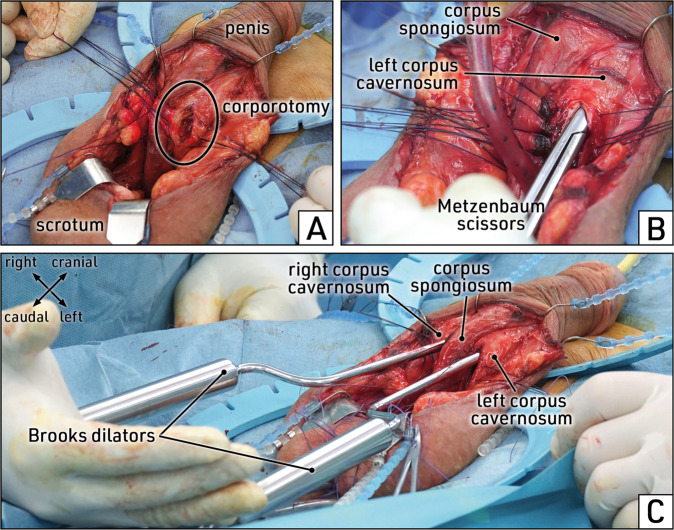
Stepwise surgery: corporotomy and corporal dilation. Latero-ventral view of the exposed penile corpora. **A** A longitudinal corporotomy is performed on the left corpus cavernosum at the correct position, and the cutting edges are temporarily retained with sutures. **B** Metzenbaum scissors are inserted into the corpus cavernosum for initial dilation while staying in close contact with its laterodorsal wall. **C** Distal dilation is completed using Brooks dilators. Images obtained during surgery.

The corporotomy for insertion of the cylinders must be located sufficiently proximal to avoid visible tubing underneath the penoscrotal skin (“Maserati penis”). SKW introduced the penile strap, which is included in several Scott self-retaining retractor systems (e.g. Coloplast, Boston Scientific, Rigicon). The top of the corporotomy should be in line with the penile strap which serves to elevate and stretch the penis. From the penile strap the corporotomy extends dorso-caudally over a distance of 1.5 cm (Fig. 6A).

#### Topographic anatomy

**Fig. 7:**
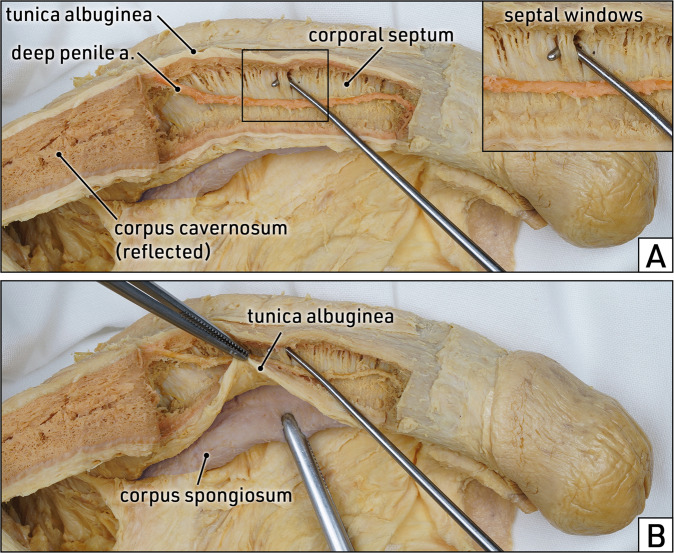
Topographic anatomy: corpora cavernosa and corpus spongiosum. Lateral view of the penile shaft. The skin and the penile fasciae are removed to expose the penile corpora. **A** The tunica albuginea of the right corpus cavernosum is incised longitudinally and reflected to open the corpus cavernosum. While the intracavernous tissue is preserved in the reflected portion of the corpus cavernosum (red area), the remaining corpus is emptied to expose the deep penile artery and the corporal septum. The insert shows multiple slit-like fenestrations (inserted hook) of the corporal septum which extend predominantly along its dorsal aspect and connect the two corpora cavernosa. **B** The ventral part of the tunica albuginea of the corpus cavernosum is lifted (upper forceps) from the corpus spongiosum/urethra (lower forceps) to illustrate its delicate and flexible consistency in this region. Formalin-fixed specimen.

In general, the wall of the corpora cavernosa is very rigid and thick compared to the corpus spongiosum. This resistant consistency must be considered during salvage IPP placement. In contrast, the ventral/paraurethral part of the corpora cavernosa is thinner and more flexible compared to the dorsolateral aspect (Fig. 7). These regional differences in rigidity should be considered during corporoplasty with or without graft in cases of reconstructive procedures for Peyronie’s disease.

### Distal dilation with first safety check

#### Surgical procedure

Dilation of the distal corpora cavernosa can be performed with standard dilators (Brooks or Hegar dilators). The primary rule for corporal dilation is gentle passage of the instrument to create sufficient space for placement of the cylinder. Quick, forceful or abrupt movements of the dilator (“quick bird movement”), especially in cases of intracorporeal obstructions, should be strictly avoided to prevent perforation of the tunica albuginea. We recommend starting dilation by first passing Metzenbaum scissors to create an intracorporeal tunnel and then continue dilation by using the dilator instruments (Fig. 6B, C). During the entire dilation procedure, care must be taken to guide the instruments along the lateral wall of the corpora cavernosa and thus avoid urethral injury or distal crossover of the cylinders.

**Fig. 8:**
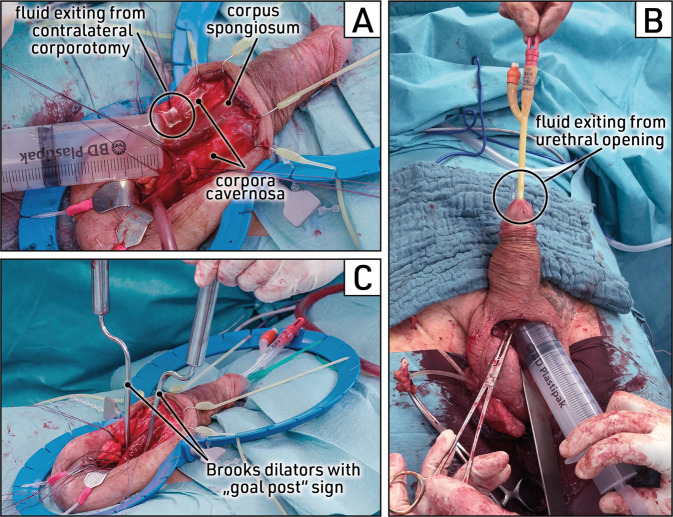
Stepwise surgery: safety checks. **A** Saline solution is injected with a syringe into the left corpus cavernosum via the corporotomy. The fluid should exit through the contralateral corporotomy via the fenestrated corporal septum. **B** In case of distal urethra perforation during corporal dilation, the fluid enters the urethra and is discharged from the urethral opening around the catheter. **C** Brooks dilators are inserted into dilated proximal corpora cavernosa and should align at the same angles, planes and depths, forming a “goal post” sign. Images obtained during surgery.

The first safety check to rule out distal urethral perforation is carried out after corporal dilation has been completed. The corpus cavernosum is irrigated via the corporotomy. Due to the fenestrated corporal septum, fluid is expected to exit through the opposite corporotomy (Fig. 8A). In case of urethral perforation, fluid will be discharged from the urethral opening around the catheter (Fig. 8B).

#### Topographic anatomy

The weakest part of the corporal tunica albuginea is located adjacent to the penile urethra (Fig. 7B). To avoid urethral injury, during corporal dilation the instruments should be directed against the laterodorsal aspect of the corpora cavernosa and not passed in medioventral direction. Moreover, subtle dissection of the corpora cavernosa illustrates that the corporal septum is not an impermeable continuous wall but is equipped with multiple slit-like windows (Fig. 7A). Therefore, the fenestrated corporal septum is prone to injury when the dilation instrument is moved too medially, thus creating a tunnel from one corpus cavernosum to the other and the risk of cylinder crossover.

### Proximal dilation and second safety check

#### Surgical procedure

Proximal dilation of the corpora cavernosa is also performed via the corporotomy using the same dilation instruments. Again, it is crucial to avoid forceful movements and follow the inferior pubic ramus in laterodorsal direction until the shaft of the dilator contacts the pubic bone. When proximal dilation is completed, the corresponding safety check is performed by inserting both dilators into the proximal corpora. Correct dilation is indicated by a “goal post” sign formed by the two dilators aligned at equal angles, planes and depths (Fig. 8C). A missing “goal post” sign could be evidence of proximal corporal perforation or crossover dilation.

#### Topographic anatomy

**Fig. 9:**
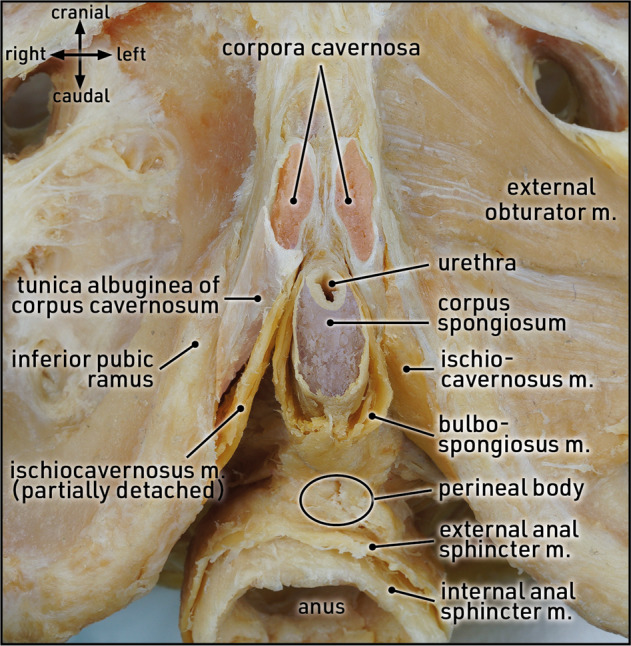
Topographic anatomy: proximal penile corpora. Caudal view of the perineal region. The mobile part of the penis is sharply cut at the level of the pubic symphysis; fatty and fascial tissues are completely removed. The corpora cavernosa (red) are ensheathed by the tunica albuginea, attached to the inferior pubic ramus, and covered with ischiocavernosi muscles. On the right side the ischiocavernosus muscle is partly detached from the proximal part of the corpus cavernosum to illustrate its conical narrowing. The proximity of the corpora cavernosa, both to the corpus spongiosum/urethra and the anal sphincters/anorectum, is clearly discernible. Formalin-fixed specimen.

The proximal corpora cavernosa are covered by the ischiocavernosi muscles and closely attached to the inferior pubic ramus (Fig. 9). As it courses along the inferior pubic ramus, proximal dilation must be carried out by following the pubic bone in laterodorsal direction. Moving the dilation instruments too medially may cause injury to the anal sphincter complex or the rectum itself, as these structures are close to the proximal corpora cavernosa. Moreover, the proximal segments of the corpora cavernosa are characterized by a continuous conical narrowing with an acute-angled tip at its end. Thus, care must be taken to gently dilate the terminal narrow part of the proximal segment in order to avoid corporal perforation.

## Discussion

### Anatomical studies related to IPP implantation

Detailed anatomical studies have a decisive impact on the successful implementation of complex surgical procedures [15]. However, we have just a handful of anatomical studies dedicated to improving IPP implantation which have addressed and explored the following anatomical peculiarities:

Hsu et al. [5] studied the composition of the corporal wall and showed that the tunica albuginea is a bi-layered connective tissue structure, except for the ventral groove adjacent to the urethra where only the inner layer is present. Due to its reduced strength, this paraurethral part of the tunica albuginea is particularly prone to perforating injuries during corporal dilation and corresponds to the area in which penile fractures most frequently occur. It was concluded that corporal dilation should be carried out by staying at the lateral side of the corpora cavernosa in order to prevent distal urethral perforation.

In line with these considerations, Pagano et al. [10] described intracavernosal pillars dividing the corporal space into dorsomedial and ventrolateral compartments. Based on these findings, the authors recommend corporal dilation and subsequent cylinder placement preferably into the dorsomedial compartment to avoid damage to the urethra and the ventral tunica albuginea.

Shafik et al. discovered the variable presence of a triangular corpora-glans ligament that may direct the tips of the cylinders beneath the glans. If the cylinders are too short to reach the glans penis, the absence of this ligament may cause penile deformities including the true floppy glans syndrome [6, 16].

Regarding the sensory innervation of the penile glans, Kozacioglu et al. [8] found a highly variable numbers of main and terminal branches of the dorsal penile nerve entering the glans. In particular, the presence of perforating branches emerging from the inferior aspect of the dorsal penile nerve was regarded as a limiting factor during mobilization of the dorsal penile NVB. The variable penile neuroanatomy also explains why some patients still suffer from a spotty loss of sensation after uneventful surgical mobilization of the dorsal penile NVB.

Henry et al. performed a large study to explore the topographic anatomy related to retropubic reservoir placement [7]. While the mean distance between the superficial inguinal ring and the filled urinary bladder was 2.61 cm, it increased to 6.45 cm when the bladder was empty. Moreover, the mean distance of the external iliac vein was only 3.23 cm from the piercing site of the transversalis fascia. Thus, the authors recommended evacuation of the urinary bladder before retropubic reservoir placement in order to avoid lateral deep dissection into the retropubic space and prevent injury to major vessels and visceral organs.

If the retropubic space is obliterated due to previous pelvic surgery, such as radical prostatectomy or cystectomy, high submuscular placement of the reservoir might be an option to minimize the risk of severe complications [17, 18]. However, Ziegelmann et al. [9] showed in body donors (*n* = 10, penoscrotal approach with bilateral reservoir placement), that despite the surgeon´s best intentions and no perioperative uncertainty, the final location of the reservoirs varied considerably: 16 reservoirs (80%) were located anterior to the transversalis fascia, 1 (5%) retroperitoneal, and 1 (5%) intraperitoneal. Thus, to avoid intraperitoneal reservoir location, the surgeon may perform an additional incision under direct visual control for correct high submuscular placement of the reservoir [19].

This study on surgical anatomy related to IPP implantation aims to complement these previous valuable contributions. Based on our experiences gained from body donor workshops and anatomical insights obtained from pre-dissected specimens, we learned the following lessons which may help to further facilitate the procedure and avoid complications:

- A transverse and centrally applied skin incision slightly below the penoscrotal junction is optimal to preserve scrotal skin innervation.
- Proper deployment of the self-retaining retractor and hooks ensures complete exposure of the proximal corpora cavernosa and facilitates correct proximal dilation.
- Use of the penile strap elevates and puts the penis on stretch, so that the penile fasciae can be easily dissected from the corpora cavernosa for optimal exposure.
- The corporotomy should be performed proximal to the penoscrotal junction by a longitudinal paraurethral incision in order to prevent injury to perineal nerve fibers and the penile urethral bulb.
- When corporotomy is to be performed by infrapubic approach, care must be taken to preserve the dorsal penile NVB which forms a condensed trunk in this region.
- As the dorsal penile NVB spreads into multiple intermingled nerve fiber strands in a horsetail-like pattern, its complete elevation may cause hypesthesia of the glans penis even if done correctly.
- Dilation of the distal corpora cavernosa should be performed by guiding the instruments (scissors followed by dilators) along the lateral aspect of the tunica albuginea, because its ventral aspect is less rigid and close to the urethra.
- Perforation of the urethra can be demonstrated by a safety check. In the event of perforation, fluid instilled into the corpora cavernosa is discharged from the urethral opening.
- Due to multiple fenestrations, the corporal septum may easily be perforated during dilation or cylinder insertion and lead to a crossover position of the cylinders.
- Dilation of the proximal corpora cavernosa should follow the inferior pubic ramus to which the proximal corpora cavernosa are attached in order to avoid corporal perforation or injury to the anal sphincter complex/rectum.
- Due to the conical narrowing of the proximal corpora cavernosa, dilation should be carried out gently without using “quick bird movements”, and should result in a “goal post” sign.

## Conclusions

A successful IPP implantation depends on several factors, such as the patient´s comorbidities, BMI, previous pelvic/penile surgeries, configuration of the external genitalia, the surgeon’s level of skill, technical familiarity with the IPP device, and timing of the surgical procedure. However, in all instances, an essential prerequisite for safe IPP implantation is in-depth knowledge of the topographic anatomy and anatomic peculiarities of the structures being addressed during the surgical procedure. A thorough review of the relevant anatomical landmarks is recommended in order to achieve an optimal surgical outcome, and is particularly important when embarking on an IPP implantation program.

## Data Availability

All data supporting the reported results are archived in the Institute of Anatomy, Christian-Albrechts University Kiel, Germany. Publicly archived datasets were not analyzed or generated during the study.
